# Electronic cigarette extract induced toxic effect in iPS-derived cardiomyocytes

**DOI:** 10.1186/s12872-020-01629-4

**Published:** 2020-08-05

**Authors:** Hesham Basma, Swetha Tatineni, Kajari Dhar, Fang Qiu, Stephen Rennard, Brian D. Lowes

**Affiliations:** University of Nebraska Medical Center, Omaha, NE USA

## Abstract

**Background:**

Cigarette smoking is an important risk factor for cardiac diseases. In the current study, we sought to assess the effect of electronic cigarette extract (ECE) and conventional cigarette smoke extract (CSE) on cardiomyocytes.

**Methods:**

iPSCs-derived cardiomyocytes were used in the study to evaluate cellular toxicities. Cells were exposed to either ECE or CSE for two consecutive days as an acute exposure or every other day for 14 days. Concentration of nicotine in both ECE and CSE were measured by Mass-Spectrometry and Q-Exactive-HF was used to identify other ingredients in both extracts. Fluorescent microscopy was used to measure the oxidative stress after ECE and CSE exposure. Motility and beat frequency of cardiomyocytes were determined using the Sisson-Ammons Video Analysis system. Heart failure target panel genes of exposed cardiomyocytes were compared to control unexposed cells.

**Results:**

Despite nicotine concentration in CSE being six-fold higher than ECE (50 μg in CSE and 8 μg in ECE), ECE had similar toxic effect on cardiomyocytes. Both CSE and ECE generate significant cellular reactive oxygen species. The Sisson-Ammons Video Analysis (SAVA) analysis showed significant changes in myocyte function with both CSE and ECE slowing beating and increasing cell death. Chronic exposure of both ECE and CSE significantly decreased cardiomyocytes viability long term at all doses.

Target panel gene expression profiles of both ECE and CSE exposed cardiomyocytes were different from controls with distinct pattern of genes that involved cell proliferation, inflammation, and apoptosis.

**Conclusion:**

ECE and CSE produce similar cardiomyocyte toxicities which include generating oxidative stress, negative chronotropic effects, adverse changes in myocardial gene expression and ultimately cell death.

## Background

### Electronic cigarettes (E-cigarettes)

More patients die from the cardiovascular complications of smoking than from the pulmonary sequelae. Despite lack of data, electronic Nicotine Delivery Systems (ENDS) are publicly perceived an alternative safer and less obtrusive option than cigarettes leading to an increase in smoking, especially among adolescents. Many believe e-cigarettes are less harmful because ENDS generate fewer combustion products than conventional cigarettes. Currently, there are no adequate studies to support this claim or that have evaluated the toxic effect of the flavors or other components in the vapor. Smoking remains a public health crisis and the risk of death from cigarette smoking is increasing among women and the increased risks are now nearly identical for both genders [1]. Little is known about the mechanisms by which smoking or ENDS disrupt cardiovascular health and there is a need for toxicity studies [2].

ENDS may attract smokers not only in providing nicotine in an aerosolized and non-inflammable form but also in mimicking the rewarding behavioral patterns that reinforce smoking. The liquid that is vaporized in an e-cigarette is available to consumers in a wide variety of flavors, including mint/menthol, and fruit flavors. Although addition of “flavors” is prohibited in cigarettes (with the exception of menthol) by the Family Smoking Prevention and Tobacco Control Act of 2009, this practice is not currently prohibited in other tobacco products, like e-cigarettes. Retail sales data suggest that the consumption of flavored e-cigarettes and tobacco products, such as flavored cigars, has increased in recent years and recent studies show that youth and young adults may find these flavored products more appealing than their unflavored counterparts [3, 4]. Nicotine doses from e-cigarettes vary tremendously depending on characteristics of the user (experience with smoking conventional cigarettes or e-cigarettes), technical aspects of the e-cigarette, and levels of nicotine in the e-liquid. Although studies of nicotine doses in youth and young adults are lacking, studies of adults have found delivery of nicotine from e-cigarettes in doses ranging from negligible to even larger than conventional cigarettes [5–7]. Nicotine dosing studies are necessary to compare relative toxicity among the different delivery systems. Their popularity has increased dramatically, and ENDS are the most commonly used tobacco product among youth. In 2016, more than 2 million U.S. middle and high school students used ENDS in the past 30 days, including 4.3% of middle school students and 11.3% of high school students [8].

There is an urgent need to define the toxicity of these new products and understand the biology of smoking related injury. In one of the recent studies, mice exposed to nicotine from e-cigarettes have decreased cardiac fractional shortening and ejection fraction in compared to controls [9]. Animal and clinical toxicity studies are slow, expensive and raise ethical concerns. Since cardiovascular disease is the major cause of smoking related mortality, our objective was to develop a readily applicable in vitro method that could directly compare the toxicity of ENDS and conventional smoking on myocyte pathophysiology, transcriptional profiles, and survival. This can, therefore, provide a basis for practical screening of ENDS for potential toxicities.

## Methods

### Cell lines

Human iPSCs-derived cardiomyocytes were purchased from iCell Cardiomyocytes® (Cellular Dynamics, Madison, WI). These cells are highly purified, human cardiomyocytes derived from iPSCs using CDI’s proprietary differentiation and purification protocols. These cells are a mixture of electrically active. We have used these cells in previously published work [10]. Tissue culture 48-well plates were coated with sterile 0.1% gelatin and Cardiomyocytes were plated 60 min after coating. Cardiomyocytes were plated at 150,000 cells per well according to manufacturer recommendations and maintained in cardiomyocytes media that have been specially formulated to maximize the cell viability (Cellular Dynamics, Madison, WI). Under these conditions, they start to contract 2 days after plating. Cells were incubated at 37 °C for 4–5 days before treatment to allow for attachment to plate and for contraction to commence.

### Smoke exposure

iCell Cardiomyocytes were incubated with different concentrations of electronic and regular smoke extract. All experiments were done in triplicate. A commercially available eGo electronic cigarette device that uses a cylindrical transparent reservoir that holds e-liquid ready to be filled was utilized in the study. Drops of e-liquid are placed in the reservoir that contains piece of cotton connected to the heating element and atomizer. Electronic cigarette extract (ECE) was prepared by bubbling smoke from the electronic cigarette using liquid that contains 6 mg of nicotine per ml through 15 mL of deionized water at a speed of 60 cc/min for 10 min. Cigarette smoke extract (CSE) was prepared from one 84-mm cigarette (research cigarette 3R4F; University of Kentucky) without filter in the same way. The resulting solutions were considered to be 100% ECE and CSE, respectively, and filtered with a 0.22-μm pore filter (Lida Manufacturing). From the filtered 100% extract solutions, 2.5, 5, and 10% ECE and CSE solutions were prepared in cardiomyocytes media. Five hundred microliters of each were added to the respective wells in the treatment plate. ECE and CSE were added to treatment plates for two consecutive days as acute exposure for all experiments or every other day for 14 days as chronic exposure for the survival and contractility experiments. After the exposures, cells were either treated for ROX detection or trypsinized, collected, and RNA was isolated for RNA-seq analysis.

### Mass spectroscopy

Samples were diluted (100x) in acetonitrile and infused into the mass spec (4000 Qtrap) at a flow rate of 15 μl/min (*n* = 4 in each group). Additional make-up flow through LC was done at 0.1 ml/ml with mobile phase (0.1% formic acid: acetonitrile: 50:50). Standard nicotine concentrations (10 ng/ml to 1000 ng/ml) were used to optimize the technique. Nicotine was identified in ESI positive mode (m/z: 163.1). Sample concentrations were calculated by comparing peak heights of nicotine in samples with those of the standards (10 ng/ml to 1000 ng/ml). To identify other toxic substances in both CSE and ECE, samples were infused at 15 μL/min into the Q-Exactive-HF (Thermofisher Scientific) in positive ion mode for intact mass analysis. The scan range was 100 to 500 m/z. The most intense peaks were manually selected using a 0.1 m/z window and fragmented using HCD. The fragments were collected for 20 scans. The MS2 spectra were converted into mgf files using Proteome Discoverer (Thermofisher Scientific) to extract the peak m/z and intensity. The list of peaks was then submitted to the Metlin- XCMS online tool for compound identification using accurate mass of the precursor ion and the fragmentation profile.

### Video microscopy of Cardiomyocytes

The Sisson-Ammons Video Analysis (SAVA) system, which combines phase contrast microscopy and computerized frequency spectrum analysis, was utilized to measure the beat frequency of cardiomyocytes and to generate a survival curve by motility analysis [11]. Actively beating cardiomyocytes were observed and their motion quantified by counting the cardiomyocytes beat frequency (CBF) in 10 s videos and whole field analysis using SAVA. The number of motile points for each 10-s digital video image was determined using a software algorithm in SAVA. The algorithm assesses if a change in light intensity occurs in a 16-pixel zone in which each zone represents a 4 × 4-pixel area. For every 640 × 480-pixel video image, the number of motile zones is calculated from a possible 19,200 total zones. As cardiomyocytes stop beating or cells detach the number of motile points decreases over time. All data points were recorded in an Excel sheet and computed to determine the percentage of survival in chronic exposure. Contractility was determined using Fourier analysis of the field of view and all frequencies represent the mean ± 1 standard error of the mean (SEM) from 6 separate groups or fields.

### ROS and Oxidative stress measurements

Black 96-well sterile plates with clear bottoms (Corning, NY) were used: one plate for controls, and one that was treated with different concentrations of smoke extract as described previously. All experiments contained 6 wells and using the acute exposure paradigm. Levels of intracellular reactive oxygen species (ROS) were measured by using a fluorescence probe, Dihydroethidium (DHE) (sigma-Aldrich, St. Louis, MO) following the second treatment with ECE and CSE. Antimycin A ((50 μM) (sigma-Aldrich, St. Louis, MO) was used as positive control and was incubated for 60 min prior of DHE. Subsequently, all wells containing cardiomyocytes except for negative control were incubated with DHE (10 μM) for 30 min in cardiomyocytes media. Cells with and without DHE were both serve as controls for the experiment. The cells only control was used to measure cells autofluorescence. Fluorescence for ROS was measured SpectraMax i3x microplate reader (Molecular Devices, Chicago, IL) with an excitation of 518 nm and emission of 606 nm. Fluorescent images were collected and analyzed using Carl Zeiss Axio Observer fluorescence microscope and Zeiss ZEN Pro Imaging Software (Carl Zeiss, Norway).

### Targeted panel

A custom panel designed in our lab to target 140 genes that have been associated with reverse remodeling in heart failure including inflammatory, contractile and other genes. Same panel we used it in previous study but with addition of few genes to assess self-renewal and pluripotency [12]. We have utilized the same technique that we previously published [10]. Briefly, RNA was extracted from cells using RNeasy kit (Qiagen, Germantown, MD). RNA was reverse transcribed by reverse transcriptase to synthesize complementary deoxyribonucleic acid (cDNA) from 10 ng of RNA isolated form cardiomyocytes using the Ion AmpliSeq™ RNA RT Module (Thermo Fisher, Waltham, MA) and Applied Biosystems thermal cycler (Applied Biosystems, Beverly, MA). Partial digestion of primer sequences was performed using FuPa reagent from Ion AmpliSeq™ RNA Library Kit. Ion AmpliSeq™ adaptors were then ligated to the targeted deoxyribonucleic acid (DNA). Two step purification and amplification were carried out and DNA library was quantified using Qubit® 2.0 fluorometer was used with Qubit® dsDNA HS Assay Kit. Ion library (Thermo Fisher, Waltham, MA). Emulsion polymerase chain reaction (PCR) on ion sphere particles was carried out to amplify diluted libraries with the Ion One Touch™ 2 System. Sequencing was carried out for all samples on the Ion Torrent Personal Genome Machine (PGM) (Thermo Fisher, Waltham, MA).

### Statistical method

The data analysis for SAVA are based on the average cardiomyocytes beat frequency (CBF) measure from each sample in 10 s time interval. Whole field analysis was determined using Fourier analysis of the field of view and all changes represent the mean ± 1 standard error of the mean (SEM) from 6 separate groups or fields.

Analysis of oxidative stress, survival and contractility was done using paired t-test or Wilcoxon signed-rank test between two groups as appropriate with *P*-value less than or equal to 0.05 was considered statistically significant.

The R package RNA-SeqPower was used for determining the sample size necessary for the RNA-seq studies (R version 3.5.1) [13]. We conservatively conduct the power analysis assuming control and treated groups are independent with positive correlation between paired samples. Assuming a sequencing depth of 20 million, and a coefficient of variation (CV) of 0.4, α = 0.0001 to help adjust for multiple comparison, we anticipate better power than 81% in detecting a 2-fold change between two groups. The RNA-sequencing data was filtered to keep the genes with nonzero reads for at least 2 samples. After filtering there were 128 genes available for analysis. The filtered data was normalized with the method of trimmed mean of M-values (TMM) and then converted to cpm (counts per million) using edgeR (Empirical analysis of digital gene expression data in R) package in Bioconductor developed by Robinson et al. [14]. The fold change of normalized expression between each exposed sample and the control was calculated. Genes were identified to be potentially differentially expressed between two conditions using cutoff of 2-fold change. Pathway analysis were evaluated for identified genes using Ingenuity Pathway Analysis (IPA, QIAGEN, Redwood City, CA).

## Results

### Nicotine concentrations and mass spectroscopy analysis

**Fig. 1:**
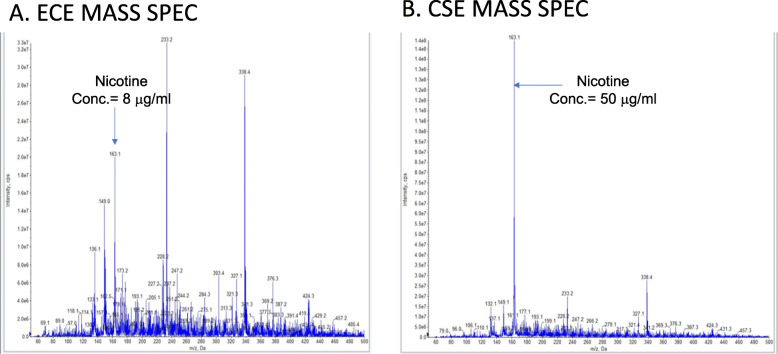
Mass Spectrometry analysis of ECE and CSE. **a**. ECE Mass Spec profile showing a concentration of 8 μg/ml of nicotine and other peaks that represents other ingredients. **b**. CSE Mass Spec profile with a concentration of 50 μg/ml nicotine. Nicotine standard was used to calculate the nicotine concentration in the different patches

**Fig. 2:**
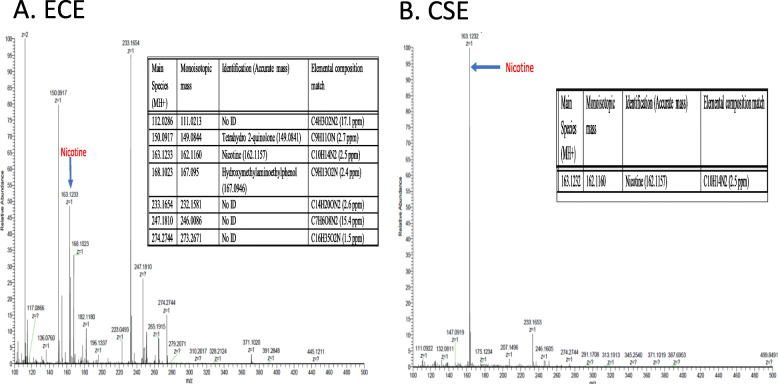
Q-Exactive-HF mass spec analysis of ingredients in ECE and CSE. Ingredients identified in **a**- ECE batch using Q-Exactive-HF in positive ion mode for intact mass analysis. **b**. Nicotine as the major peak identified in four different batches of CSE

In order to determine the concentration of nicotine in all batches of prepared ECE and CSE, mass spectroscopy was utilized using a nicotine standard of 1 mg/ml. Nicotine concentration in the different fresh batches of CSE averaged 50 μg/ml (*N* = 4) and for ECE was 8 μg/ml (N = 4) (Fig. 1 show a representative CSE and ECE mass spec analysis chart). CSE was six-fold higher than ECE. It is clear from the representative analysis chart that nicotine was the main peak in CSE while ECE was more complex and contained multiple peaks. These data prompted us to further analyze the other peaks using Q-Exactive-HF in positive ion mode for intact mass analysis. Metlin- XCMS online tool for compound identified several compounds, listed in Table 1A and Fig. 2, were identified in ECE but not in CSE including but not limited to, tetrahydro2-qinolone that was found in 2 batches of ECE (2 and 4). Two peaks were identified all analyzed ECE patches with no ID (111.0213 and 264.0086 respectively). In contrast the CSE analysis as shown in Table 1B and Fig. 2 revealed that the major peak was nicotine. Both identified and unidentified substances found in ECE may contribute to the harmful effect produced in cardiomyocytes.

**Table 1:** A. Ingredients identified in ECE batch and B. CSE batch using Q-Exactive-HF in positive ion mode for intact mass analysis. Several ingredients were identified in ECE. B. Nicotine is the only major peak identified in four different batches of CSE

Sample name	Main Species (MH+)	Monoisotopic mass	Identification (Accuratemass)	Elemental composition match
A				
ECE 1	112.0286	111.0213	No ID	C4H3O2N2 (17.1 ppm)
	163.1233	162.1160	Nicotine (162.1157)	C10H14N2 (2.5 ppm)
	247.0159	246.0086	No ID	C7H6O8N2 (15.4 ppm)
ECE 2	112.0287	111.0213	No ID	C4H3O2N2 (17.1 ppm)
	150.0917	149.0844	Tetrahydro 2-quinolone 149.0841)	C9H11ON (2.7 ppm)
	163.1233	162.1160	Nicotine (162.1157)	C10H14N2 (2.5 ppm)
	233.1654	232.1581	No ID	C14H20ON2 (2.6 ppm)
	247.1811	246.0086	No ID	C7H6O8N2 (15.4 ppm)
	265.1916	264.1843	Tetracaine (264.1838)	C15H24O2N2 (2.3 ppm)
ECE 3	112.0286	111.0213	No ID	C4H3O2N2 (17.1 ppm)
	163.1233	162.1160	Nicotine (162.1157)	C10H14N2 (2.5 ppm)
	247.0160	246.0086	No ID	C7H6O8N2 (15.4 ppm)
ECE 4	112.0286	111.0213	No ID	C4H3O2N2 (17.1 ppm)
	150.0917	149.0844	Tetrahydro 2-quinolone (149.0841)	C9H11ON (2.7 ppm)
	163.1233	162.1160	Nicotine (162.1157)	C10H14N2 (2.5 ppm)
	168.1023	167.095	Hydroxymethylaminoeth ylphenol (167.0946)	C9H13O2N (2.4 ppm)
	233.1654	232.1581	No ID	C14H20ON2 (2.6 ppm)
	247.1810	246.0086	No ID	C7H6O8N2 (15.4 ppm)
	274.2744	273.2671	No ID	C16H35O2N (1.5 ppm)
B				
CSE 1	163.1232	162.1160	Nicotine (162.1157)	C10H14N2 (2.5 ppm)
CSE 2	163.1232	162.1160	Nicotine (162.1157)	C10H14N2 (2.5 ppm)
CSE 3	163.1233	162.1160	Nicotine (162.1157)	C10H14N2 (2.5 ppm)
CSE 4	163.1232	162.1160	Nicotine (162.1157)	C10H14N2 (2.5 ppm)

### Reactive Oxygen Species (ROS)

**Fig. 3:**
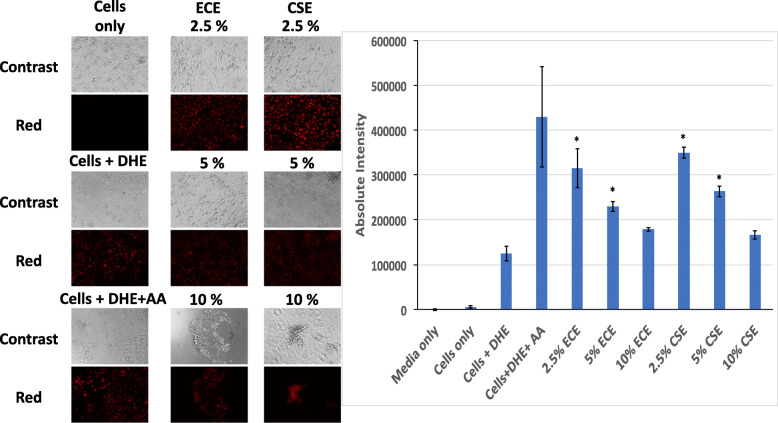
Oxidative stress and Reactive oxygen species (ROS) produced by ECE and CSE. **a**. Florescence images of significant increase in reactive oxygen specious produced by different concentrations of ECE and CSE compared to control cells (*P* < 0.05). **b**. Absolute intensity of DHE florescence in cardiomyocytes exposed to different smoke concentrations of ECE and CSE and in positive control. (* is significantly different from control at *p* < 0.05)

Normal heart cardiomyocytes were exposed to different concentrations of ECE and CSE for two consecutive days. Fluorescence for ROS was measured, as shown in Fig. 3, there was a significant and concentration dependent increase in reactive oxygen species when cells were exposed to ECE and CSE compared to control cells (*P* < 0.05). At 2.5% ECE and CSE, the Mean ± SEM of absolute intensity were 3.15E05 ± 4.32E04 and 3.5E05 ± 1.2E04 respectively with a significant difference to control (Cells +DHE) of 124E05 ± 1.6E04. There was no significant difference between CSE and ECE induction of ROS at similar concentrations even though the nicotine concentration in ECE was significantly less than CSE. Other byproducts in ECE may have contributed to producing high ROS. Positive control, Antimycin A produced ROS with absolute intensity of 4.9305 ± 1.1E04. Cells started dying at 5% ECE and CSE exposure as evidenced by regular, florescence microscopy. This phenomenon increased at 10% exposure (Fig. 3a). These results indicate that acute exposure at both 5 and 10% of ECE and CSE was toxic to the cells and it is consistent with our published data for lung fibroblasts exposed to 10% CSE [15].

### Cellular motility analysis

**Fig. 4:**
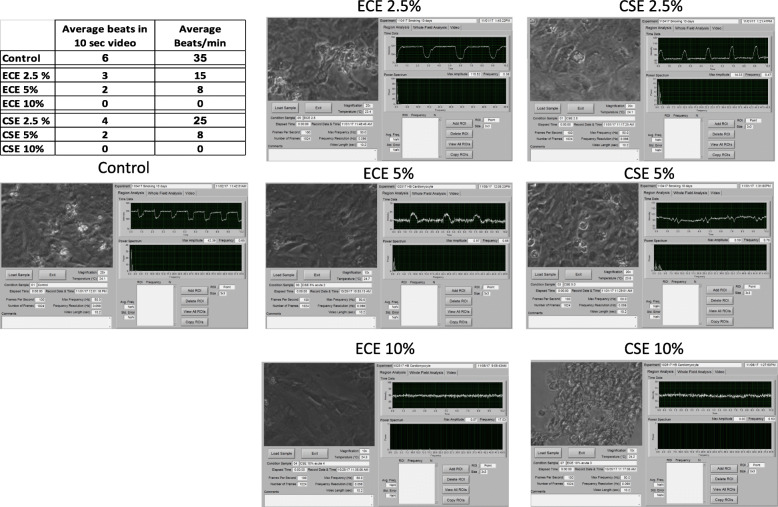
Whole field analysis and survival for cardiomyocytes in control and chronic ECE and CSE smoke exposure. Control cardiomyocytes had 75% of the cells beating at day 14 and 47% beating at day 21 and the cells survived to day 28 with 12% beating. Cells exposed to 5% exposure, cells survived for 14 days in CSE and for in ECE. While 10% of ECE and CSE exposed cells were drastically affected and cells stopped beating completely 7 days days. Both ECE and CSE 2.5% exposed cardiomyocytes beat at 47, 42, 27 and 1% for ECE and 45, 42,24 and 1% for CSE in days 7,14,21,24 respectively

**Fig. 5:**
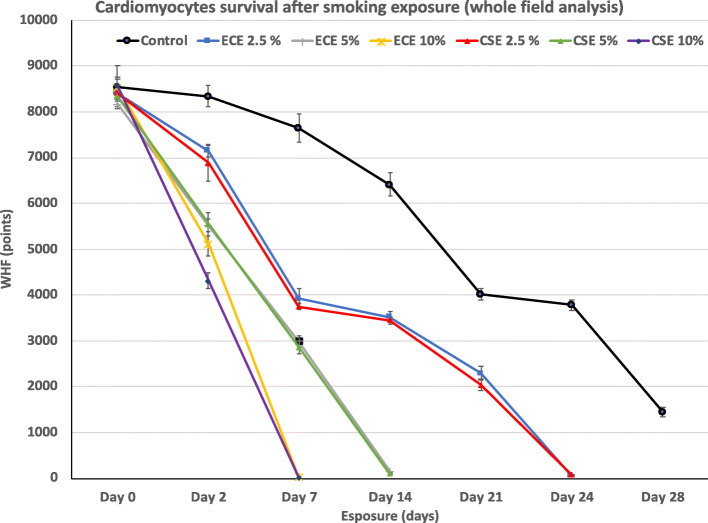
Cardiomyocytes contractility in control, ECE and CSE acute smoke exposure. In acute exposure (2 consecutive days), control cardiomyocytes beat 35 beats/min while 2.5% ECE slowed beating to 15 beats/min. In contrast 2.5% CSE beat 25 beats/min. Exposure to ECE and CSE at 5% CSE made the cells beat at lower rate and stopped at 10% CSE

We utilized the SAVA system that was originally used to determine cilia beat frequency [11] to determine acute and chronic effects on cellular chronotropy and viability with different extracts. For cardiomyocytes, average beat frequency in 10 s time interval and whole field analysis were used to determine cellular effects. As shown in Fig. 4, significant beat frequency differences occurred in control compared to exposed cells. In acute exposure, control cardiomyocytes beat 35 beats/min while 2.5% ECE slowed beating to 15 beats/min. In contrast 2.5% CSE slowed to 25 beats/min (*p* < 0.05). As noted above, at 5 and 10% of ECE exposure disrupted the cell integrity and stopped beating. Acute exposure for 5% CSE made the cells beat at a lower rate and the was completely inhibited at 10% CSE. Cells tended to form clumps at 10% ECE and CSE. To determine the long-term exposure effect of smoking on cardiomyocytes, whole field analysis (WFA) and survival curve was done by computing all the numbers of chronic exposure WFA after every exposure and was compared to control non-exposed. As shown in Fig. 5, control cardiomyocytes had 75% of the cells beating at day 14 and 47% beating at day 21 and the cells survived to day 28 with 12% beating. In contrast, both ECE and CSE 2.5% exposed cardiomyocytes beat at 47, 42, 27 and 1% for ECE and 45, 42,24 and 1% for CSE in days 7,14,21,24 respectively. Cells exposed to 5% exposure, cells survived for 14 days in CSE and for in ECE. While 10% of ECE and CSE exposed cells were drastically affected and cells stopped beating completely 7 days days. Different biological effects of ECE and CSE on cardiomyocytes were summarized in Table 2.

**Table 2:** A. Fold change in genes significantly down-regulated in both ECE and CSE exposed cardiomyocytes compared to control. B. Fold change in genes significantly upregulated in both ECE and CSE exposed cardiomyocytes compared to control. All genes were significant with *p < 0.05*

	GENE ID	ECE	CSE
A	**Genes down-regulated in both ECE and CSE**		
1	ABCC9	0.3877	0.3689
2	ATRNL1	0.1308	0.0517
3	CAV3	0.3877	0.4919
4	KCNIP2	0.1795	0.5124
5	LIN28A	0.2019	0.0576
6	MYLK	0.5287	0.3354
7	NFKB2	0.4936	0.5358
8	NPPA	0.1497	0.3978
9	PHF2	0.4102	0.3930
10	SGCD	0.1710	0.0543
11	SLC2A4	0.3151	0.3487
12	SMAD2	0.4206	0.4507
13	TNNI3	0.4889	0.4740
14	TNNT2	0.3351	0.3970
15	ZNF704	0.4648	0.2270
B	**Genes upregulated in both ECE and CSE**		
1	NPPB	2.9595	5.2738
2	PABPC1L	8.4004	2.7669
3	PER3	5.8156	4.6115
4	TMEM43	2.5847	4.1859
5	WEE1	6.2772	2.8987

### Gene expression analysis and Ingenuity Pathway Analysis (IPA)

RNA-seq for HF target panel designed in our lab was performed on control, 2.5% ECE and 2.5% CSE smoked cardiomyocytes. After filtering there were 128 genes available for analysis. Compared to control, 30 and 44 genes were down-regulated in ECE and CSE exposed cardiomyocytes respectively by more than 2-fold. In addition, 18 and 21 genes were upregulated in ECE and CSE respectively compared to control by more than 2-fold. Of these genes 15 were down-regulated and 5 up-regulated in both ECE and CSE compared to control (Table 3). These altered genes involved inflammation, cell proliferation, regeneration and apoptosis. Consistent with our previously published data, many of these gene including Myosin light chain kinase (MYLK), Natriuretic Peptide B (NPPB) and Troponin I3 (TNNI3) are essential for normal heart function and response to stress [12].

**Fig. 6:**
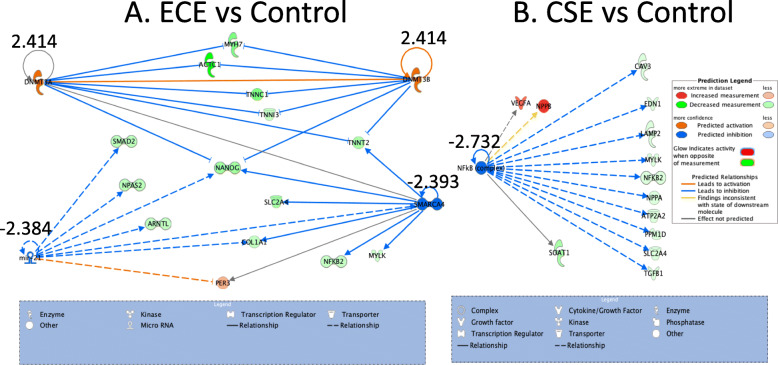
Ingenuity pathway analysis of the differentially expressed genes in ECE and CSE compared to controls. IPA of the differentially expressed genes in cardiomyocytes exposed to ECE and CSE compared to control with significant Z-scores for each pathway. **a** Upregulation of DNA methylation signal, downregulation of MIR-21 and SMARCA4. **b** downregulation of NFKB signal in CSE exposed cardiomyocytes

IPA analysis of the differentially expressed genes in cardiomyocytes revealed different results when ECE or CSE were compared to control. In ECE there was a significant increase Z-scores in upstream methylation signals (2.414 for both DNMT3A and B pathway). Mir21 and SMARCA4 were downregulated with Z-scores of − 2.384 and − 2.393 respectively. On the other hand, comparing CSE to control lead to only a significant down regulation of the NFKB pathway way with a Z-score of − 2.732. However, when both ECE and CSE were compared to control only SMARCA4 was significantly downregulated but no other upstream pathways were significantly up or down regulated (Fig. 6).

**Table 3:** Summary of ECE and CSE effects on cardiomyocytes. Overall comparison between ECE and CSE effects on cardiomyocytes was summarized in the table

Biological Effect	ECE	CSE
**Average Nicotine conc.**	8 μg/ml	50 μg/ml
**Reactive Oxygen Specious in 2.5%**	3.15E05 ± 4.32E04	3.5E05 ± 1.2E04
**Survival in day 7,14,21,24 for 2.5%**	47%, 42%, 27%, 1%	45%, 42%,24%, 1%
**Significant down regulated genes compared to control**	30	44
**Significant up regulated genes compared to control**	18	21

## Discussion

In this study we compared conventional cigarette smoke extract and e-cigarette extract on cardiomyocytes at the functional level in terms of reactive oxygen species production, chronotropy, survival, and at the molecular level by RNA-seq. The main finding of this study is that ECE produces toxic effects on cardiomyocytes similar to CSE and does so at lower equivalent exposure of nicotine. ESE markedly increases production of reactive oxygen species by cardiomyocytes, has negative chronotropic effects, alters myocardial gene expression, and decreases cellular survival. In the Health eHeart Study by Wang et al., E-cigarette use alone was associated with risk of health symptoms and conditions. They concluded that in dual users of conventional cigarette and e-cigarette, the use of e-cigarettes was not associated with less exposure to tobacco smoke or health risks [16]. On the other hand, Kim et al. reported that dual users had greater nicotine dependence and higher urinary cotinine levels than cigarette-only smokers [17].

In order to assess the toxic effect of the two different methods of smoking in our study, we sought to determine the nicotine concentration and other different ingredients in these smoke extracts. In our study we found that the concentration of nicotine in CSE was significantly higher than ECE by six-fold. The average of nicotine in the different batches of ECE was 8 μg/ml, low compared to previous studies where the average nicotine content in different 54 e-cigarette products was 11 mg/ml [18]. The difference between our study and Hahn et al. is that we measured the nicotine in the extract after bubbling the e-liquid which we believe is a more physiological approach to testing the extract. In another clinical study done by Yan et al., they found that nicotine plasma concentrations after 1.5 h of product use were significantly lower in the users of e-cigs than of regular cigarettes [19]. We also identified some other ingredients in e-cigarettes that were not found in the standard research cigarettes. This included tetracaine, an anesthetic, which is possibly being added to these products to increase tolerability. The liquid contents of electronic nicotine delivery systems contain numerous components that potentially are toxic including aldehydes, nitrosamines, alkaloids, metals, and over 7000 unique flavors such as bubble gum and chocolate. New assays are needed to better evaluate the safety and toxicity of these additives as this industry is poorly regulated.

Reactive oxygenation species are generated as part of normal physiology but excessive production leads to cell death through both apoptosis and necrosis [20]. Oxidative stress is also known to promote telomere shortening and dysfunction. Inhaled pollutants from the air or cigarette smoke are capable of causing cardiovascular toxicity via direct effects of absorbed compounds or indirect effects from lung injury and inflammatory mediators or alterations in autonomic regulation and oxidative stress potentiates all these mechanisms [21]. Our results indicate that ECE and CSE produce similar effects on cellular oxidative stress which is deeply concerning as recent observational data suggesting that E-cigarette exposure is associated with a marked increase in myocardial infarction, stroke, and circulatory problems [22]. This is in accordance with Espinoza-Derout et al. in their mice study where they reported an increased oxidative stress and mitochondrial DNA mutations in mice treated with e-cigarette [9].

When e-cigarettes are accompanied by a measurable increase in plasma nicotine concentration, it increases heart rate, and diastolic BP [7, 23, 24] likely through modification of autonomic regulation. A tobacco-industry study suggested that the acute increases in heart rate and blood pressure that followed tobacco-cigarette use were greater than those that followed e-cigarette use [19]. In contrast, direct exposure of both ECE and CSE in our study had negative chronotropic effects, slowing beating cardiomyocytes and leading to cell death in a dose dependent fashion. There are no adequate human studies to support the cardiovascular safety or toxicity of different ENDS or the commercial products that are available for smoking. The effects of diverse additives including flavors or the actual components in the vapor, carriers such propylene glycol and glycerin, as well as other substances such as benzoic acid, have not been determined. Our results suggest that ENDS are at least as harmful as standard cigarettes.

The molecular mechanisms of smoking related toxicity on human myocytes are largely unknown due to difficulties in obtaining heart tissue from smokers. This study utilized a targeted panel of genes focused on known abnormalities that occur in heart failure to evaluate the direct toxicity of different nicotine delivery systems on human cardiomyocytes [12, 25–27]. Targeted gene expression analysis suggests broad transcriptional changes are induced by these products contributing to injury or adaptive responses to the stress. These transcriptional changes are related to contractile proteins, metabolism, inflammation and potassium channels. IPA pathway analysis in our study revealed that DNA methylation in upstream signaling of ECE treated cardiomyocytes was upregulated. DNA methylation is an important epigenetic regulator of gene expression and this process is likely contributing to many of these changes in gene expression. This is in concordance with two studies done in mouse and human cells [28, 29]. This is also consistent with our previous study revealing that TGF-β induces similar effect in cardiomyocytes [10]. While CSE treated cardiomyocytes downregulated NFKB pathway in contrary to a study indicated that CS induced NF-kappaB in lymphocytes through IKK activation, I-kappa-B-alpha degradation, and the reduction in the intracellular glutathione levels [30].

The potential clinical implications of the current study are substantial. Our results indicate that e-cigarettes are as toxic to cardiomyocytes as conventional cigarettes. ENDS use is reaching an epidemic stage especially with teenagers that will likely contribute to long-term adverse cardiovascular events in addition to other pulmonary diseases and cancer.

Study Limitations: The study only measured the direct toxicities of electronic and conventional smoke extracts to cardiomyocytes. It is not feasible or ethical to conduct large scale in humans due to the toxicities and addictive potential of these agents. Long term epidemiologic studies will be necessary to determine the public health consequences of ENDS. Studies to address these toxicities in peripheral blood and aiming of finding a marker to detect the degree of the toxicity to ENDS should be conducted. This study is also limited in that it only measured a targeted number of genes previously associated with heart failure. These agents likely cause much broader changes in transcriptional profiles. Using iPSCs-derived cardiomyocytes was essential in our study because accessing and maintaining primary human cardiomyocytes is difficult. Ultimately, using iPSCs-derived cardiomyocytes will allow for broader studies of cellular toxicities and transcriptional profiles along with the mechanisms that control these changes.

## Conclusion

ECE and CSE produce similar cardiomyocyte toxicities which include generating oxidative stress, negative chronotropic effects, adverse changes in myocardial gene expression and ultimately cell death. Cardiomyocytes derived from iPS cells provide an informative and convenient model to evaluate the emerging epidemic of alternative nicotine delivery systems.

## Data Availability

Available upon request.

## Abbreviations

ECE: Electronic cigarette extract
CSE: Cigarette smoke extract
ENDS: Electronic nicotine delivery systems
ROS: Reactive oxygen species
iPSCs: Induced pluripotent stem cells
